# The Latin American version of the internalized stigma of mental illness scale (LA-ISMI): a multicentric validation study from three Latin American countries

**DOI:** 10.1186/s12955-019-1238-2

**Published:** 2019-11-27

**Authors:** Alejandra Caqueo-Urízar, Alfonso Urzúa, Anderson Loundon, Mohamed Boucekine, Guillaume Fond, Laurent Boyer

**Affiliations:** 1 Instituto de Alta Investigación, Universidad de Tarapacá, 1520 Antofagasta, Arica Chile; 2 Universidad Católica del Norte, Avda. Angamos, 0610 Antofagasta, Chile; 3 Aix-Marseille Univ, EA 3279 – Public Health, Chronic Diseases and Quality of Life - Research Unit, 13005 Marseille, France

**Keywords:** Internalized stigma, Schizophrenia, Psychometric properties, Validity

## Abstract

**Background:**

To date, no data have been available concerning the psychometric characteristics of the Internalized Stigma of Mental Illness scale (ISMI-29) in Latin American countries. The aim of this study was to validate a Latin American version of the ISMI in people with schizophrenia.

**Methods:**

The study included 253 stabilized outpatients with schizophrenia from 3 Mental Health Services in three Latin American countries: Bolivia (*N* = 83), Chile (*N* = 85) and Peru (*N* = 85). We analyzed the psychometric properties using item response and classical test theories. An item reduction was then performed to improve the psychometric properties of the ISMI-29. The final version of the ISMI was tested for construct validity, reliability, external validity and differential item functioning (DIF).

**Results:**

The five-factor structure of the ISMI-29 was not confirmed using confirmatory factor analysis (RMSEA = 0.12, CFI = 0.77, and WRMR = 2.20). Seventeen items were discarded to obtain a satisfactory psychometric version. The ISMI-12 evaluates 3 dimensions: social stigma (4 items), stigma experience (4), and self-stigma (3). The factor structure accounted for 68% of the total variance. Internal consistency was satisfactory. The scalability was satisfactory, with INFIT statistics within an acceptable range. In addition, the results confirmed the absence of DIF and supported the invariance of the item calibrations between countries.

**Conclusion:**

The ISMI-29 is not valid in our sample and should not be used in Latin American countries. The ISMI-12 is the first internalized stigma questionnaire with satisfactory psychometric properties available in Latin American countries. Its brevity could facilitate its dissemination and use in clinical settings.

## Background

People with mental illness are exposed to the negative stereotypes of the general population about people with mental illness and may internalize them, which yields the so-called internalized stigmas [1, 2]. The findings of a systematic review by Gerlinger and colleagues [3] indicate that from one-third to one-half of patients with schizophrenia (SZ) feels shame, embarrassed, guilty, and inferior to those without mental illness [4–7]. Also, as a consequence of the disorder, patients with internalized stigma are more likely to experience depression, reduced self-esteem, reduced recovery orientation, reduced empowerment, and increased perceived devaluation and discrimination [8–12]. Self-stigma is a barrier for early antipsychotic treatment onset and appropriate treatment in general, because the patients tend to lose their motivation to receive medical health care [13–16]. Another consequence is the increased duration of untreated psychosis has been associated with worse prognosis and also with higher hospitalization, involuntary admission and suicidal behaviour [17]. These last elements impact on the therapeutic effect, because, as Chang and colleagues [18] pointed out is expected to have better outcomes when health care professionals take into not only the symptomatology but also self-stigma in persons with mental illness [16, 18, 19].

However, the consequences of self-stigma do not only affect the patient, but also the caregiver. This type of stigma is called “courtesy stigma”, being stigmatized because of one’s relationship to a person with a stigmatizing mark [18, 20, 21] and “affiliate stigma”, internalizing the stigma because of the relationship that affects the caregiver’s self-esteem and burden [7, 20, 22, 23].

Accurate and appropriate assessment of internalized stigma is thus critical to reduce duration of untreated psychosis to improve medical-social programs and to guide public health policies for SZ people. The Internalized Stigma of Mental Illness scale (ISMI-29) is one of the most widely used measurements of internalized stigma in mental health research [11, 24–26]. There is only one validation in Spanish, but it was carried out in Spain, whose version obtained good values of internal consistency and test-retest reliability, for the total score of the scale (0.91 and 0.95 respectively), as well as for the five subscales, except for the Stigma Resistance subscale (Cronbach’s alpha 0.42) [27]. To date, the psychometric characteristics of the ISMI-29 have not been explored in Latin American countries [9]. Even though, the language is similar, we cannot exclude substantial socio-cultural and economic differences between Spain and Latin American countries, influencing the phenomenon of stigmatization. In addition, the previous study did not report how the factorial structure described in their samples fit the initial structure of the tested instrument, which remains a key point when considering validity. Restricted data regarding validity and reliability were also provided. For these reasons, the extent to which SZ patients in Latin American countries can validly self-report their internalized stigma using the ISMI-29 is a crucial issue that has not been sufficiently explored. Furthermore, shortening the ISMI-29 could make the assessment on internalized stigma efficiently.

The aims of this study were thus to validate a Latin American version of the ISMI in people with schizophrenia and to shorten the ISMI-29 into a brief measure to efficiently measure internalized stigma.

## Methods

### Study participants

Overall, 253 stabilized SZ outpatients were consecutively recruited between May 2012 and February 2013 in the three public ambulatory psychiatric care centers of three areas: Arica, northern Chile (*N* = 85, 33.6%), Tacna, southern of Peru (*N* = 85, 33.6%), and La Paz, Central-Western of Bolivia (*N* = 83, 32.8%). The three centers shared similar characteristics in terms of size, type of treatment delivered to patients, professionals and free access of care.

#### Inclusion criteria

All stabilized community-dwelling patients diagnosed with schizophrenia according to the criteria of International Classification of Diseases (ICD), 10th version [28] were included in this study.

#### Exclusion criteria

Patients with history of neurological disorders (including stroke, epilepsy and head injury) or all illnesses affecting central nervous system were not included in the present study.

### Procedures

The study was approved by the Ethics Committee of the University of Tarapacá and the National Health Service of Chile. Two psychologists, who were part of the research team, trained for scale evaluation, and supervised by the principal researcher (AC-U), conducted the evaluations of the participants under the auspices of the mental health services of each country. The length of time of the evaluation was between 20 and 30 min.

Before the start of the survey, written informed consent was requested and received from the patient. The objectives of the study were explained as well as the voluntary nature of participation. No compensation was offered for participating in the study.

### Data collection

#### Recruitment process

At each center, during a three- month window, all patients were invited to participate as they came for their monthly follow-up visits. The overwhelming majority of the patients agreed to participate.

#### Demographic and illness characteristics variables

Age, gender, ethnicity (Aymara and non-Aymara), educational level (≥ 12 years or <  12), employment status (unemployed or employed), family income (measure of the total salary per month for all members of the family, expressed in US dollars), age at onset the disorder, the number of hospitalizations in the last 3 years were reported. All patients were administered antipsychotics. The presence or absence of add-on integrated treatment (defined by psychotherapy, family psychoeducation, and/or day care hospital in addition to pharmacological treatment) was also reported.

Concerning ethnicity, the Aymara is the largest ethnic group in the region, with a population of 2 million people, and has lived in the Andes Mountains for centuries. Recent generations of Aymara have undertaken a massive migration from rural towns to large cities and, thus, receive healthcare services from the same clinics as non-Aymara individuals [29–31]. This ethnic group share a particular worldview were the concept of mental health disorder is understood according to this worldview. Therefore, although there are differences in socioeconomic terms between countries, there are a number of cultural traditions that unify them.

### Instruments

#### Internalized stigma [11].

The ISMI scale is a self-rated assessment of the subjective experience of stigma for people with mental illnesses that comprises 29 items across five subscales: alienation (6 items), stereotype endorsement (7 items), discrimination experience (5 items), social withdrawal (6 items), and stigma resistance (5 items). The alienation measures the subjective experience of being less than a full member of society or having a ‘spoiled identity’. The stereotype endorsement measures the degree to which respondents agree with common stereotypes about people with mental illness, such as ‘mentally ill people tend to be violent’ and ‘I can’t contribute anything to society because I have a mental illness.’ The discrimination experience subscale intends to capture respondents’ perception of the way that they currently tend to be treated by others, such as ‘People ignore me or take me less seriously just because I have a mental illness’ and ‘People discriminate against me because I have a mental illness.’ The social withdrawal contains items, such as ‘I don’t talk about myself much because I don’t want to burden others with my mental illness’ and ‘I avoid getting close to people who don’t have mental illness to avoid rejection.’ The Stigma Resistance intends to portray the experience of resisting or being unaffected by internalized stigma [11].

Each item is rated on a 4-point Likert scale ranging from 1 = strongly disagree to 4 = strongly agree with higher scores indicating higher internalized stigma. Each score is calculated by adding the item scores together and then dividing by the total number of answered items. A high total score on the ISMI scale indicates more severe internalized stigmatization. The Spanish version of the ISMI-29 was used in this work [27]. Following the method used by Lysaker and colleagues [25], 4 categories can be created for each score: 1.00–2.00 (minimal to no internalized stigma), 2.01–2.50 (mild internalized stigma), 2.51–3.00 (moderate internalized stigma), and 3.01–4.00 (severe internalized stigma). Following the method used by Ritsher & Phelan [7], 2 categories can also be created for each score: 1.00–2.50 (does not report high internalized stigma), and 2.51–4.00 (reports high internalized stigma).

#### Positive and Negative Syndrome scale for Schizophrenia (PANSS) [32].

This 30-item, 7-point (1–7) rating scale has been specifically developed to assess psychotic symptoms in SZ individuals with five factors (positive, negative, cognitive, depressive and excitement subscores). The PANSS has been translated and validated in Spain by Peralta and Cuesta [33] and in Mexico by Fresán, et al. [34]. The psychometric properties were satisfactory with principal component analysis explaining 53.4% of the total variance and Cronbach’s alpha > 0.8 for each dimension [34]. This scale was administered by the treating health professionals.

#### Schizophrenia Quality of Life Questionnaire (SQoL18) [35].

Quality of life (QoL) was assessed using the SQoL18, a self-administered QoL questionnaire designed for people with schizophrenia [35] and validated in Latin America [36]. QoL score ranges from 0, indicating the lowest QoL, to 100, the highest QoL. Factor analysis performed in the 3 countries (Bolivia, Chile and Peru) showed that the questionnaire’s structure adequately matched the initial French structure of the SQoL18. The unidimensionality of the dimensions was preserved, and the internal/external validity indices were close to those of the reference population [36].

### Statistical analysis

Socio-demographic and clinical characteristic descriptions were done with frequencies and percentages for categorical variables and with means and standard deviations for continuous variables.

The 5-factor structure of the ISMI-29 was verified using confirmatory factor analysis (construct validity). In this confirmatory factor analysis, we used the diagonally weighted least squares (DWLS) method to estimate the coefficients and fit indices. The following indicators were required to confirm that this structure match with our data. The Root Mean Square Error of Approximation (RMSEA) is acceptable if < 0.08, the Comparative Fit Index (CFI) is higher than 0.9, and the Weighted Root Mean Square Residual (WRMR) (a fit index generated from the use of DWLS) is lower than 0.9. Considering previous validation studies in different countries [9], we hypothesized that the 5-factor structure initially developed would not fit with our data. In this case, the following reduction procedure will be applied.

The construct validity was assessed using principal component factor analyses with varimax rotation [37], in order to determine a new structure and the number of independent dimensions of internalized stigma. Eigenvalues greater than or equal to 1 were retained [38]. Descriptive statistics were performed to examine the response distribution to each item and dimension. The items with the following characteristics were removed: low response rate (< 20%), low index discrimination (< 0.70), decrease of cronbach’s alpha coefficients, and multiple loading (> 0.4) of an item on several factors [39]. Once the structure and the number of items are fixed, this final version was tested for construct validity, reliability, external validity and acceptability.

Item-internal consistency (IIC) was assessed by correlating each item with its scale (corrected for overlap) using Pearson’s coefficient (correlation of 0.4 recommended for supporting item-internal consistency) [40]; item discriminant validity was assessed by determining the extent to which items correlate more highly with the dimensions they are hypothesized to represent than with the other ones [41]. For each dimension scale, internal consistency reliability was assessed by Cronbach’s alpha coefficient (coefficient of at least 0.7 expected for each scale [40].

The uni-dimensionality of each dimension was assessed using Rasch analysis. The goodness-of-fit statistics inlier-sensitive mean square (infit MnSq), ranging between 0.7 and 1.3 ensured that all items of the scale measured the same concept. Floor and ceiling effects were reported assessing the homogeneous repartition of the response distribution. Differential item functioning (DIF) analyses were performed, which compared the item differences between groups of individuals according to socio-demographic parameters (gender, ethnicity, and country) to check whether all items behave the same way [42]. The DIF means that an item performs and measures differently for one subgroup of a population than for the other.

The external validity was assessed by studying relations between dimensions of ISMI scores and demographic, illness and QoL characteristics. The underlying assumption was that stigma was associated with being a man and Aymara, lower family income, higher psychologic symptoms and lower quality of life [43]. We also explored the relations between the new version of ISMI and ISMI-29 scores. The underlying assumption was that total and dimension scores of the new ISMI would be highly correlated with ISMI-29 and also more correlated with scores of similar dimensions.

All the tests were two-sided. Statistical significance was defined as *p* < 0.05. The statistical analyses were performed using the SPSS version 20.0 software package (SPSS Inc., Chicago, IL, USA) and Mplus Software.

## Results

### Sample characteristics

Two hundred and fifty-three SZ patients were enrolled in this study. The sociodemographic and clinical features are listed in Table 1.

**Table 1:** Socio-demographic and clinical characteristics of the study sample (*N* = 253)

Patients	Mean ± SD, median [IQR] or n (%)*
Age in years	35.6 ± 12.5
Gender	
Women	83 (33.6)
Men	164 (66.4)
Ethnicity	
Non-Aymara	136 (53.8)
Aymara	117 (46.2)
Educational level	
≥ 12 years	40 (15.8)
< 12 years	213 (84.2)
Employment status	
With employment	78 (31.2)
Without employment	172 (68.8)
Monthly family income (US dollars)	331.3 [144.9; 517.9]
Age at onset of the disease	20.9 ± 6.4
Number of hospitalizations	1 [2; 0]
Type of mental health treatment	
Integrated	31 (12.3)
Only pharmacological	222 (87.7)
Symptoms severity	
PANSS total score	71.3 ± 28.2
Positive factor	8.3 ± 4.6
Negative factor	18.6 ± 8.4
Depressive factor	6.4 ± 3.7
Cognitive factor	7.3 ± 4.0
Hostile-excitement factor	11.5 ± 5.9
Quality of life	
S-QoL 18 index	54.3 ± 14.4

*Mean ± SD: mean ± standard deviation; median [IQR]: median [Inter Quartile Range]; *n* (%): effective (percentage)

*PANSS* Positive and Negative Syndrome scale for Schizophrenia

*S-QoL18* Schizophrenia Quality of Life questionnaire

### Construct validity of the ISMI-29

The five-factor structure of the ISMI-29 was not confirmed using confirmatory factor analysis: RMSEA = 0.12, CFI = 0.77, and DWLS = 2.20. Cronbach’s alpha coefficients were not satisfactory (discrimination experience = 0.47, social withdrawal = 0.68, and stigma resistance = 0.67), except for two dimensions (alienation = 0.73, and stereotype endorsement = 0.72).

### Item reduction of the ISMI-29

Seventeen items were discarded for the following reasons: 5 items for low index discrimination, 12 items were deleted after examination of items’ structure using the principal component analyses (4) and Cronbach’s alpha coefficients (8). The final version contained 12 items (ISMI-12).

### Construct validity, internal structural validity and reliability of the ISMI-12

The results are summarized in Table 2. The structure of the ISMI-12 was confirmed by principal component factor analysis, identifying a 3-factor structure accounting for 68% of the total variance. The dimensions were named according to their constitutive items: social stigma (4 items), stigma experience (4 items), and self-stigma (3 items). The 12 items are detailed in the Appendix. Internal consistency was satisfactory for all dimensions: each item achieved the 0.40 standard for item-internal consistency. The correlation of each item with its contributive dimension was higher than with the others (item discriminant validity). Floor effect ranged from 16.6 to 27.9% and ceiling effect from 19.5 to 29.2%. Cronbach’s alpha coefficients ranged from 0.77 to 0.88, indicating satisfactory internal consistency. The overall scalability was globally satisfactory: no items showed an infit MnSq outside the acceptable range except for 2 items and country. Dimension and index scores was then calculated. The results also confirmed the absence of DIF according to gender, ethnicity, and country and supported the invariance of the item calibrations.

**Table 2:** Dimension characteristics of the ISMI

Dimension/index (number of items)	M (SD)	Missing values %	Item-internal consistency (min-max)	Item discriminant validity (min-max)	Floor %	Ceiling %	Alpha^a^	INFIT^b^ (min-max)
Dimension 1: social stigma (4)	2.8 (0.9)	0.8	0.67–0.77	0.24–0.46	16.6	29.2	0.88	0.82–1.25
Dimension 2: stigma experience (4)	2.5 (0.8)	0.6	0.55–0.61	0.32–0.44	24.3	19.5	0.77	0.85–1.08
Dimension 3: self-stigma (3)	2.4 (1.0)	0.7	0.67–0.75	0.24–0.47	27.9	21.9	0.85	0.87–1.19
Index (12)	2.6 (0.7)	0.7	NA^c^	NA^c^	NA^c^	NA^c^	0.88	NA^c^

^a^Cronbach’s Alpha, ^b^Rasch’s statistics, ^c^NA Not Applicable

M (SD) mean (standard deviation); a higher score represents a higher level of unawareness

According Lysaker, et al., (2007) [25], 23.4% (57) of individuals had minimal to no internalized stigma, 27.0% (66) had mild internalized stigma, 25.0% (61) had moderate internalized stigma, and 24.6 (60) had severe internalized stigma. Following the method used by Ritsher & Phelan, 2004 [24], 50.4% (123) did not report high internalized stigma, and 49.6% (121) reported high internalized stigma.

### External validity of the ISMI-12

The results are summarized in Table 3. Higher psychotic symptomatology, lower quality of life, and lower family income were associated with internalized stigma. Men had higher social withdrawal than women, Aymara had higher alienation than non-Aymara, and employment was associated with lower internalized stigma.

**Table 3:** External validity of the ISMI dimension scores and index

	Dimension 1: social stigma	Dimension 2: stigma experience	Dimension 3: self-stigma	Index
Symptoms severity				
PANSS total score	0.26**	0.33**	0.24**	0.33**
Positive factor	0.24**	0.30**	0.22**	0.30**
Negative factor	0.31**	0.24**	0.18**	0.30**
Depressive factor	0.18**	0.26**	0.19**	0.27**
Cognitive factor	0.13*	0.27**	0.22**	0.23**
Hostile-excitement factor	0.01	0.18**	0.13*	0.12
Quality of life				
S-QoL 18 index	−0.38**	−0.47**	−0.42**	−0.51**
Age (years)	0.08	0.11	−0.07	0.04
Monthly family income (US dollars)	−0.21**	− 0.11	−0.14*	− 0.21**
Age at onset of the disease (years)	−0.02	− 0.07	−0.04	− 0.07
Number of hospitalizations	0.09	0.04	0.02	0.05
Gender				
Men	2.9 (0.8)	2.4 (0.8)	2.4 (1.0)	2.6 (0.7)
*p*-value	**0.009**	0.499	0.865	0.303
Ethnicity				
Aymara	2.8 (0.9)	2.5 (0.7)	2.6 (0.9)	2.7 (0.7)
*p*-value	0.220	0.590	**0.011**	0.072
Education level				
< 12 years	2.8 (0.9)	2.5 (0.8)	2.5 (1.0)	2.6 (0.7)
*p*-value	0.177	0.106	0.068	0.062
Employment status				
With employment	2.5 (0.9)	2.2 (0.8)	2.2 (0.9)	2.3 (0.7)
Without employment	2.9 (0.8)	2.6 (0.8)	2.5 (1.0)	2.7 (0.7)
*p*-value	**0.001**	**0.001**	**0.016**	**< 0.001**
Mental health treatment				
Only pharmacological	2.8 (0.9)	2.4 (0.8)	2.4 (1.0)	2.6 (0.7)
*p*-value	0.630	0.460	0.354	0.803

*PANSS* Positive and Negative Syndrome scale for Schizophrenia

*S-QoL18* Schizophrenia Quality of Life questionnaire

Bold values *p* < 0,05, *p < 0.05, **p < 0.01

The correlations between the scores of ISMI-12 and ISMI-29 are presented in Table 4. All the dimensions were significantly correlated (r ranged from 0.18 to 0.97). The total scores of ISMI-12 and ISMI-29 were highly correlated (*r* = 0.93). The 3 dimensions of the ISMI-12 (i.e., social stigma, stigma experience, and self-stigma) were highly correlated with three similar dimensions of the ISMI-29 (social withdrawal *r* = 0.98, discrimination experience *r* = 0.97, and alienation *r* = 0.92).

**Table 4:** Correlations between the scores of ISMI-12 and ISMI-29

ISMI-12 ISMI-29	Dimension 1: social stigma	Dimension 2: stigma experience	Dimension 3: self-stigma	Index
Alienation	.439^**^	.616^**^	.921^**^	.787^**^
Stereotype endorsement	.346^**^	.522^**^	.580^**^	.574^**^
Discrimination experience	.546^**^	.972^**^	.522^**^	.836^**^
Social withdrawal	.983^**^	.535^**^	.352^**^	.832^**^
Stigma resistance	.176^**^	.343^**^	.315^**^	.312^**^
Total	.700^**^	.800^**^	.739^**^	.930^**^

*p < 0.05, **p < 0.01

### Acceptability

The proportion of missing values per dimension never exceeded 1.0%.

## Discussion

In this study, we have demonstrated the validity and reliability of ISMI-12 in a large multicenter sample of Latin American community-dwelling SZ patients from three countries. The ISMI-12 presents interesting characteristics for a widespread use in SZ patients in Latin America.

Although the literature shows several instruments of self-stigma measurement, within which some shorter scales are observed such as the ISMI-10 [12], the ISMI-9 [44, 45] and the Self-Stigma Scale-Short (SSS-S, 9 items) [46, 47] and others more extensive scales including the Self-stigma of Mental Illness Scale (SSMIS, 40 item) [48], the Self-stigma of mental Illness scale short form (SSMIS-SF, 20 items) [49]; the Consumer Experiences of Stigma Questionnaire (CESQ, 20 items) [50], the Depression Self-stigma Scale (DSSS, 32 items) [51], the Stigma Scale (SS, 28 items) [52], the Discrimination and Stigma Scale (DISC, 36 items) [53, 54], the ISMI-12 is the only short scale that has been valid in a Latin American context. According to several authors, a short form of a scale is frequently associated with better acceptability [55]. The average completion time is expected to be less than 5 min and this will facilitate its use in routine clinical practice.

The internal structure retrieved several important dimensions of stigma for patients. The classification of the items is different from that of the ISMI-29 and may add a complementary approach to this scale. This new classification appears transversal to the different dimensions (i.e., alienation, stereotype endorsement, discrimination experience, social withdrawal and stigma resistance) of the ISMI-29 (trans-dimensional), with a grouping more centred on the patient’s experience rather than on a theoretical and conceptual approach of the stigma. The first dimension addresses the social aspect of stigma while the ISMI-29 focused on social withdrawal. In this dimension (i.e., social stigma), items explored social dependency (item 1), social withdrawal (item 2 and 3), and social exclusion (items 4 and 5). Previous studies reported the importance of the social issue in the phenomenon of stigmatization: social anxiety, social withdrawal, and lower social functioning [3]. The second dimension is about experience of stigma including items discrimination (items 6, 7 and 9) and negative belief about the self (item 8). Previous studies reported the closely link between discrimination and the impact on self-esteem and stereotype [1]. The third dimension is related to self-stigma in accordance with the definition proposed by Corrigan and Watson (2002) [1]: an “*internalisation of public stigma*” or as “*the product of internalization of shame, blame, hopelessness, guilt and fear of discrimination associated with mental illness*”. Lastly, we can also note that the majority of items on stigma resistance were discarded, confirming that stigma resistance do not function like a subdimension of internalized stigma of mental illness construct [56].

Concerning the psychometric properties, our proposal meets standards. The internal structure was supported by a high internal consistency. Internal consistency reliabilities for the three dimensions were shown to be high (Cronbach’s alpha> 0.77). External validity, explored by the use of demographic, illness and QoL characteristics confirmed results of previous works on stigma. As expected, patients with higher psychotic symptoms had higher scores of stigmatization. Like other studies, a number of reasons are raised in relation to this point: thus, the greater the number of symptoms, the more likely it is to be the target of stigma from others, which would increase the probability of greater self-stigma, being more difficult for the patient to formulate positive beliefs about them. Conversely, if the patient has a lower internalized stigma, he / she is likely to be able to manage the symptoms more effectively, reducing its severity [25]. Higher scores of stigmatization were associated with lower quality of life. This result is consistent with previous studies showing reduced subjective quality of life mediated by perceived stigma and low self-esteem in SZ subjects [4, 57–60].

There are several limitations of this study. The sample may not be representative of the entire population of SZ patients (only public sector in medium–large cities) and more globally of the entire population in Latin America. Moreover, the patients in the study sample were mostly middle-aged males with mild disease severity and long illness duration. Confirmation is required for more miscellaneous and larger groups of patients. In particular, further work is needed to test the ISMI-12 in other important South American countries like, Colombia and Venezuela. Future studies should incorporate patients from the private sector given the marked social differences especially in Latin America. The “responsiveness” or “sensitivity to change” defined as the ability to detect a meaningful change, is a core psychometric property of a measuring instrument that we did not explore in this study. Its examination is required in future studies using longitudinal data collection.

## Conclusion

The ISMI-12 is the first internalized stigma questionnaire with satisfactory psychometric properties available for SZ individuals in Latin American countries. Its brevity may be appropriate and useful for research and clinical practices.

## Data Availability

The datasets generated and/or analysed during the current study are not publicly available due Government policy but are available from the corresponding author on reasonable request.

## Appendix

**Table 5:** Latin American version of the ISMI: ISMI-12

Items ISMI-12 (English)	Items ISMI-12 (Spanish)	Corresponding item in the ISMI-29	Dimensions
1. Because I have a mental illness, I need others to make most decisions for me.	Debido a que tengo una enfermedad mental, necesito que los demás tomen la mayoría de decisiones por mí	Item 19	Dimension 1: social stigma
2. I stay away from social situations in order to protect my family or friends from embarrassment.	Me mantengo apartado de situaciones sociales con el fin de no avergonzar a mi familia o amigos	Item 20	Dimension 1: social stigma
3. People without mental illness could not possibly understand me.	Las personas sin enfermedad mental no pueden entenderme	Item 21	Dimension 1: social stigma
4. People ignore me or take me less seriously just because I have a mental illness.	La gente me ignora o me toma menos en serio sólo porque tengo una enfermedad mental	Item 22	Dimension 1: social stigma
5. Living with mental illness has made me a tough survivor.	Vivir con una enfermedad mental me ha hecho una persona fuerte	Item 24	Dimension 1: social stigma
6. I feel comfortable being seen in public with an obviously mentally ill person.	Me siento cómodo si me ven en público con una persona que es evidente que tiene enfermedad mental	Item 14	Dimension 2: discrimination stigma
7. People often patronize me, or treat me like a child, just because I have a mental illness.	La gente a menudo tiene una actitud paternalista conmigo, o me trata como a un niño, solo porque tengo una enfermedad mental	Item 15	Dimension 2: discrimination stigma
8. I am disappointed in myself for having a mental illness.	Estoy decepcionado conmigo mismo por tener una enfermedad mental	Item 16	Dimension 2: discrimination stigma
9. People can tell that I have a mental illness by the way I look.	La gente puede decir que tengo una enfermedad mental por mi aspecto	Item 18	Dimension 2: discrimination stigma
10. I avoid getting close to people who don’t have a mental illness to avoid rejection.	Evito relacionarme con personas que no tienen enfermedad mental para evitar el rechazo	Item 4	Dimension 3: self-stigma
11. I am embarrassed or ashamed that I have a mental illness.	Estoy avergonzado de tener una enfermedad mental	Item 5	Dimension 3: self-stigma
12. Mentally ill people shouldn’t get married.	Las personas con enfermedad mental no deberían casarse	Item 6	Dimension 3: self-stigma

## Abbreviations

CESQ: Consumer Experiences of Stigma Questionnaire
CFI: Comparative Fit Index
DIF: Differential item functioning
DISC: Discrimination and Stigma Scale
DSSS: Depression Self-stigma Scale
DWLS: Diagonally Weighted Least Squares
ICD: International Classification of Diseases
IQR: Inter Quartile Ranget
ISMI: Internalized Stigma of Mental Illness scale
PANSS: Positive and Negative Syndrome scale for Schizophrenia
QoL: Quality of Life
RMSEA: Root Mean Square Error of Approximation
SQoL18: Schizophrenia Quality of Life Questionnaire
SS: Stigma Scale
SSMIS-SF: Self-stigma of mental Illness scale short
SZ: Schizophrenia
WRMR: Weighted Root Mean Square Residual
